# Temporal Tendencies: Exploring the Impact of Chronotype Timing on Youth Depression Risk

**DOI:** 10.1007/s10802-024-01287-6

**Published:** 2025-01-24

**Authors:** Dustin A. Haraden, Kathleen C. McCormick, Julianne M. Griffith, Benjamin L. Hankin

**Affiliations:** 1 Department of Psychology, Rochester Institute of Technology, 58 Lomb Memorial Drive, Rochester, NY 14623 USA; 2 College of Human Ecology, Cornell University, Ithaca, NY USA; 3 Department of Psychiatry, University of Pittsburgh School of Medicine, Pittsburgh, PA USA; 4 Department of Psychology, University of Illinois at Urbana-Champaign, Champaign, IL USA

**Keywords:** Chronotype, Youth, Depression, Puberty, Risk, Circadian Rhythm

## Abstract

Developmental changes in youth sleep preferences (chronotype) and pubertal development are consequential for youth risk for depression. Previous research has identified individual differences in chronotype in risk for psychopathology. However, little is known regarding how the *timing* of chronotype may confer risk in youth. This study addressed this gap by examining associations between chronotypal timing and symptoms of depression in youth. Community youth (*N* = 155; ${M}_{age}$=12.7) completed self-report measures of chronotype, pubertal status and depression every six months for a period of one year (three assessment points). Regression analyses showed that chronotypal timing predicted change in depressive symptoms across six months (*b* = -0.66, *p* = 0.019), but not across any other timeframe. Findings suggested that youth experiencing more of a morning preference compared to same-aged peers were at increased risk for later depression across six months. Chronotypal timing continued to predict changes in symptoms of depression controlling for gender (*b* = -0.63, *p* = 0.023) and pubertal timing (*b* = -0.72, *p* = 0.012). These findings suggest that chronotypal timing is prospectively related to changes in youth symptoms of depression. Results indicate that attention to the *timing* of normative changes in chronotype is warranted, in addition to mean-level differences.

**Supplementary Information:**

The online version contains supplementary material available at 10.1007/s10802-024-01287-6.

## Introduction

Adolescence is a time of transition, and has often been considered a particularly tumultuous period accompanied by large increases in stress related to drastic behavioral, social and biological changes (Dahl & Forbes, 2010). Throughout late childhood and early adolescence, individuals exhibit a transition in the timing and alignment of their sleep and wake patterns (i.e., circadian rhythms) in relation to the 24-h light/dark schedule (i.e., chronotype; Adan et al., 2012; Randler, 2011). On average, children show sleep/wake patterns such that they fall asleep early in the night, and also wake up early (i.e., morning preference; Roenneberg et al., 2004). During late childhood and adolescence, this pattern transitions such that the individual is more likely to fall asleep and wake *later* (i.e., evening preference; Roenneberg et al., 2004). As an individual ages, the overall alignment of their sleep/wake cycle to the light/dark schedule transitions back to rising early and going to sleep early. The shift from a morning to evening preference during adolescence is theorized to occur along with the development of puberty (Roenneberg & Merrow, 2007; Roenneberg et al., 2004). Post-pubertal youth have been shown to exhibit a later alignment (i.e., evening preference) when compared to peers who had not yet completed pubertal development (Haraden et al., 2017).

Although a mean-level transition towards an evening preference occurs during the developmental transition of adolescence, there are individual differences in the alignment of sleep/wake cycles to the light/dark schedule (i.e., chronotype), with some individuals having a later or earlier alignment than most others of a similar age. Such individual differences have been linked to the development of psychopathology. Those with a later alignment (i.e., an evening preference; going to bed and rising late) tend to experience more negative outcomes such as difficulties with physical well-being (Roenneberg et al., 2012; Urbán et al., 2011) and increases in experiences of poor mental health (Alvaro et al., 2014; Chiu et al., 2017; Haraden et al., 2017, 2019; Jankowski et al., 2019; Klimstra et al., 2010; Merikanto et al., 2013) relative to those with an earlier alignment.

Although existing work has established the importance of individual differences in overall preference for morningness/eveningness in predicting mental health outcomes, less work has examined individual differences in the *timing* of the developmental transition of chronotype during adolescence – that is, *when* the shift from morningness to eveningness occurs relative to one’s peers. The current project aimed to (1) explore the timing of chronotype development during late childhood and early adolescence, and (2) examine the role of chronotypal timing in conferring risk for depression.

### Chronotype – Circadian Rhythms

The world operates within a series of rhythms. From the 24-h period of light and dark, the menstrual cycle, to the four seasons, these rhythms exist within both biological and social contexts (Albrecht, 2012; Hlastala & Frank, 2006). Individuals experience an internal biological rhythm that closely follows the 24-h schedule of light and dark periods from day to day. However, genetic variations result in variability in how close/disparate an individual’s internal circadian rhythm is aligned to the typical daily light/dark schedule. The internal human circadian rhythm, when allowed to “free-run” (i.e., under conditions of isolation and constant light), operates on a cycle that is slightly longer than the 24-h day (approximately 24 h and 11 min ± 16 min; (Czeisler, 1999). This results in a continuous synchronization (entrainment) that must take place in order to align these rhythms as closely as possible. The internal circadian rhythm is entrained to the external light/dark cycle through environmental cues (“zeitgebers”; translates to “time giver”). Zeitgebers are consistent markers that exist within the individual’s environment such as timing of light exposure, meal times and even common periods of social interaction. The strongest zeitgeber (the highest efficiency of entrainment) for humans is light/dark exposure, and the variability in the timing of these zeitgebers results in an overall circadian preference/timing of sleep and wakefulness to the light/dark schedule (i.e., chronotype; Aschoff, 1967; Roenneberg et al., 2019).

Chronotype is organized on a continuum with the extremes being a morning preference (“morning lark”) and evening preference (“night owl”; Adan et al., 2012). An individual who has a preference for morning would tend to go to bed and rise early, whereas an individual who has a preference for evening would tend to go to bed and rise late, with most individuals falling somewhere in between these extreme tendencies. Individual differences in chronotype relate to individual differences in diverse facets of mood and behavior (for overview, see Chauhan et al., 2023). For example, individuals with an evening type are most productive and alert during the later parts of the day, while individuals with a morning type are most productive and alert in the earlier part of the day (Adan et al., 2012; Freeman & Hovland, 1934). Developmental models highlight that mean-level change in chronotype is observed across the human lifespan (Merikanto et al., 2012; Roenneberg & Merrow, 2007). Children (e.g., pre-pubertal) exhibit a greater preference for morning (rising early and going to bed early). During adolescence, these preferences normatively change such that youth demonstrate increasing preferences toward evening, beginning to rise and go to bed later. Across adulthood and older adulthood, individuals slowly transition back toward a morning preference, waking earlier and going to bed earlier (Roenneberg et al., 2004). The developmental shift in chronotype during adolescence appears to align with pubertal development/hormonal changes (Hagenauer & Lee, 2012; Morssinkhof et al., 2024) and may differ across genders, with some studies finding no difference in gender and others identifying female participants having more of an evening preference (Carskadon, 2011; Carskadon et al., 1993; Crowley et al., 2018; Fischer et al., 2017; Roenneberg et al., 2004). As such, there is a large amount of between-persons variability in youth’s chronotype within any given age group.

Individual differences arise among chronotype due to variations in the way in which the internal rhythms (i.e., body temperature and hormone secretion) becomes aligned with the external 24-h light/dark schedule (Taylor & Hasler, 2018). Variability in chronotype is present and can be related to the youth’s sex, personality, as well as environmental factors such as season of birth and the current latitude and longitude of the individual (Chauhan et al., 2023; Natale et al., 2009; Randler, 2008, 2011; Randler et al., 2015). This entrainment between internal and external clocks/rhythms impacts individuals’ ability to effectively navigate the world. For example, adolescents are predisposed to have an evening preference, but societal expectancies require early rising to engage with school. Therefore, youth often have a discrepancy between their internal/biological preference for sleep timing and the timing of the activities in their life (Roenneberg et al., 2019). Such a discrepancy can result in an accumulation of sleep debt throughout the week, and the need to “catch-up” on sleep during the weekend or “free days” (days with no social obligations). This phenomenon is known as “social jet lag” and is a measure to reflect circadian rhythm disruption (Wittmann et al., 2006).

Social jet lag commonly impacts shift workers and especially children and adolescence attending a traditional school setting. Disruptions in circadian rhythms have been shown to have negative effects on both physical and mental health, particularly feelings of loneliness and decreased satisfaction (Lyall et al., 2018; Taylor & Hasler, 2018). An individual may be experiencing social jet lag, which is normative during adolescence, but may have an earlier or later chronotype in comparison to their same-aged peers. This provides an additional layer of desynchrony between biological (internal circadian rhythms), societal (school timing) and social (earlier/later chronotype compared to same aged peers) rhythms that may result in greater impacts to mental health.

Although there is work establishing the shift in chronotype from morning to evening preference across the adolescent transition, there is limited understanding surrounding the timing at which youth begin this normative developmental process. It may be the case that there are large amounts of variability within the overall timing of this transition, similar to puberty, another socially-salient, biologically-based developmental process. Identifying youth who are advanced or lagging in their shift in chronotypal development may yield important insights into risk for psychopathology. Yet, individual differences in chronotypal timing and their association with psychopathology have not yet been investigated.

Chronotypal *timing* refers to the individual differences that arise in the timing of the transition in chronotype during adolescence. Although previous literature has examined individual differences in chronotype as they relate to psychopathology (see Walker et al., 2021), finding that individuals who hold an evening preference are at greater risk for psychopathology (Alvaro et al., 2017; Haraden et al., 2017; Urbán et al., 2011), to our knowledge, no studies have investigated associations between psychopathology and chronotypal timing. As it is currently studied, chronotype is often operationalized as a static score indicating where an individual falls on the continuum between morningness and eveningness, which, while informative, neglects to acknowledge the role of additional developmental and societal factors associated with the chronotypal transition as they relate to youth outcomes (Chauhan et al., 2023). That is, this operationalization of chronotype fails to account for whether an individual’s chronotype is at an expected level given that individual’s age, which would be expected to have important implications for youth adjustment and adaptation. Chronotypal development can be classified as “on time” when it falls close to the average relationship between chronotype and age (i.e., expected development). This reflects that an individual’s chronotype is developing in a similar way to their same-aged peers. Deviations from this expected developmental trajectory indicates “off time” chronotypal development, such that youth are “delayed” or “advanced” in their chronotype. A delayed (or negative) chronotypal timing would characterize an individual who is experiencing more of a morning preference compared to their same aged peers, whereas an advanced (or positive) chronotypal timing would reflect an individual with a greater evening preference compared to their similarly aged peers.

In a recent review, Chauhan and colleagues (2023) posited a multidimensional model to better characterize the construct of chronotype in a holistic manner, by taking into account individual, environmental and social factors. The authors criticized the current measurement of chronotype (i.e., using the Morningness-Eveningness Questionnaire (MEQ; Horne & Ostberg, 1976)or the Munich-Chronotype Questionnaire (MCTQ; Roenneberg et al., 2003)) due to their suboptimal psychometric properties, inconsistent scoring methods and cutoffs, as well as reliance on the existence of a structured and traditional work/school week and weekend (Chauhan et al., 2023). In addition, Chauhan et al. (2023) highlighted that these scales fail to take into account the impact of demographic and socio-cultural aspects when examining chronotype. The authors identify the importance of these factors and suggest that future research should take these into account when seeking to further understand chronotype. The current investigation may be one of the first step in the direction in contextualizing chronotypal development in terms of co-occurring social developmental processes, as represented by age.

### Importance of Developmental Timings

Accounting for timing into developmental/biological processes has been emphasized in a number of different research traditions, which collectively suggest that the *timing* of an event/risk, in addition to mean-level differences, has differential impacts on well-being (e.g., adversity/stress timing and psychopathology; Gunnar et al., 2009; Koss & Gunnar, 2018; Pearson et al., 2015; van den Bos & Westenberg, 2015). In considering developmental windows of particularly heightened susceptibility to environmental inputs, puberty has been identified as a particularly sensitive period as it is a defining process in an individual’s lifetime, as it indicates the transition into adulthood as well as the ability to reproduce (Ellison et al., 2012). The pubertal developmental process within humans is accompanied by changes not only in physical maturation, but also alterations in the social landscape youth inhabit and their capacity for advanced cognitive processes. This period is characterized by ongoing development of brain structure (Lenroot & Giedd, 2010), brain function (Somerville et al., 2010), cognitive capacity/executive function (Luna et al., 2010) as well as sleep (Carskadon, 2011; Feinberg & Campbell, 2010) and circadian rhythms (Carskadon et al., 1993; Roenneberg et al., 2004).

Pubertal development as a process has high levels of variability, and research has continued to develop in order to capture these individual differences. The most common and well-documented approach is to examine *pubertal timing* (Susman & Dorn, 2009). Youth who experience early pubertal timing are physically maturing *earlier* than their same aged peers, whereas youth who are “late”, tend to physically mature at a later age. There has been a historical narrative and theory (“gendered deviation hypothesis”; (Brooks-Gunn et al., 1994; Ge et al., 2006) that *early* pubertal timing for girls, and *late* pubertal timing for boys, puts youth at risk for psychopathology and impacts their overall well-being. Additional theories and empirical studies have promoted the “maturational disparity hypothesis” which suggests that any type of deviation (early or late pubertal timing) for youth is a vulnerability as it places them in difficult social and physical situations with their peers (Petersen et al., 1988). Research has focused on the changes that occur during the pubertal transition, which include psychosocial factors such as the role of physical maturation in the context of a larger social community (Carter et al., 2011; Hamlat et al., 2020; Hoyt et al., 2020; Klopack et al., 2020; Seaton & Carter, 2018). Therefore, it is important to continue to explore this developmental time period within the larger context of other social and biological changes as it appears that there is a commonality in which deviation from the *expected* process enhances the likelihood of experiencing psychological distress.

Together, this body of work clearly highlights the importance of focusing on the timing of key developmental processes, in addition to mean-level differences in the staging of these processes. As a similarly critical developmental process rooted in biology and contextualized in light of the rich social ecologies in which youth are embedded, the timing of the chronotypal transition merits similar attention. The “social zeitgeber theory” aims to capture the integration between various rhythms and purports that disruptions to mood are a result of an instability among the physical and social rhythms for an individual (Ehlers, 1988). Therefore, when youth experience a change in their physical rhythm (i.e., shift in chronotype) that may be discrepant to their social rhythm (i.e., similarly aged peers’ social ecosystem), they may be at risk for psychopathology, specifically mood disorders. Following from the social rhythms theory (Ehlers, 1988), it may be hypothesized that having a delayed preference (greater morning preference compared to peers), may result in youth being less likely to engage with peers socially during normative times (later in the evening) due to low levels of alertness and a greater likelihood of being asleep. This may lead to greater social isolation, interpersonal stress, hopelessness and symptoms of depression. On the other hand, having an advanced preference (greater evening preference compared to peers), may allow the youth more time for rumination during the evening hours after social interactions likely occur, or encourage the youth to affiliate with peer groups that are older and more likely to be engaging in risky behaviors. This may also lead to increases in interpersonal stress, parental conflict, social jetlag and symptoms of depression. Overall, deviations from the expected developmental transition of chronotype may predispose youth to risk via the harmful impact of asynchronous social and biological rhythms on mental health problems.

### Current Study

The current study sought to examine the emergence of individual differences in the development of chronotype during adolescence (i.e., chronotypal timing), and the associations between individual differences in *chronotypal timing* and symptoms of depression.

#### Aim 1: Examine the individual differences in timing of the shift in chronotype (i.e., chronotypal timing)

The development of chronotype has been previously treated as a uniform process such that all youth transition from morningness to eveningness at the same rate from childhood into adolescence. Informed by previous work in pubertal development and timing, the present work aimed to advance knowledge by examining chronotypal timing as a unique and meaningful construct that may predict key outcomes of interest. The assumption that this process occurs at similar ages for youth was directly investigated through the identification of the timing variable.

#### Aim 2: Investigate the association between chronotypal timing and depressive symptoms

Further, the present study investigated concurrent and prospective relations between chronotypal timing and symptoms of depression among youth to assess whether off-time chronotypal timing (i.e., having a more morningness or eveningness chronotype compared to same aged peers) is associated with increased risk for symptoms of depression. Gender differences were examined across the relationship between chronotypal timing and depressive symptoms. As this is the first investigation of the construct of chronotypal timing, this study was conceptualized as exploratory, and no a priori hypotheses were made regarding expected patterns of effects. Sensitivity analyses were also conducted controlling for pubertal timing to examine the extent to which chronotypal timing predicts unique variance in youth depressive symptoms after accounting for this other salient co-occurring developmental process.

## Methods

### Participants

Participants included 155 youth ($${M}_{age}$$= 12.7, 51% girls, 49% boys) who participated in a larger longitudinal study examining the emotional and social development among youth during ages captured in traditional middle school (grades 6 – 8) located within a micro-urban city in the Midwest (Griffith & Hankin, 2021). Inclusion criteria included enrollment in grades 6–8 and access to a personal internet enabled smartphone device. Baseline data included in the current project were collected from January 2019 through March 2021. Participants were predominantly white/European American (70.8%), with smaller amounts of individuals identifying as multiracial (13.6%), Asian (7.1%), Black/African American (3.9%) and other racial background (4.6%). Additionally, 5.5% of the sample identified as Latine. The majority of participating caregivers (83.9%) had completed a 4-year bachelor’s degree or higher and had a median family income between $90,000 – $179–000 (see Griffith & Hankin, 2021 for more details regarding sampling and participant characteristics). Measures for the current study were collected during 3 timepoints spaced approximately 6 months apart. During each assessment period, youth completed questionnaires about pubertal status (PDS), chronotype (MCTQ) and symptoms of depression (CDI). All study procedures were approved by the Institutional Review Board at the University of Illinois at Urbana-Champaign prior to data collection.

### Measures

The **Munich Chronotype Questionnaire** (MCTQ; Roenneberg et al., 2003; see www.thewep.org/documentations/mctq) assessed chronotype through youth self-report on various questions of sleep behavior and routines. Participants were instructed to identify their average sleep and wake cycles (e.g., “What time do you usually try going to sleep?” and “What time do you wake up in the morning?”) separately for a school-day (a day with structured commitments) and a non-school-day (a day free of structured commitments). Youth then entered estimates as to what time each of these behaviors occurred. When participants included a range of time, the researchers included the midpoint of the range as their entry. At times, participants included phrases (e.g., “When I get tired.”) that were unable to be updated resulting in a missing variable despite their participation at that timepoint. Roenneberg and colleagues (2003) suggested to use free days sleep and wake times (i.e., days without social obligations; the weekend), as opposed to school-days, in the calculation of youth’s chronotype to avoid contamination of social influences (such as school schedules and expectations of rise times) on the construct of chronotype. Thus, chronotype was operationalized as the mid-sleep point on free days ($MSF= Sleep Onset+ \frac{Sleep Duration}{2}$) (Roenneberg et al., 2003, 2012a). Additionally, to take into account “oversleep” or “catch-up sleep” that may occur on free days when youth will sleep extended periods of time, the mid-sleep point on free days *sleep corrected* was also calculated by calculating the difference of youth sleep duration on free days and weekdays (${MSF}_{sc}=MSF-(\frac{Sleep Duration:free days-Sleep Duration:weekdays}{2}$). This calculation accounts for the discrepancy between how much sleep individuals get during days with obligations and free days. The mid-sleep point on free days sleep corrected (MSF_sc_) is intended to “clean chronotype of the confounder of sleep debt” to offer an unbiased metric of chronotype (Roenneberg et al., 2019). Both variables were retained for the analyses to examine differences that may emerge when accounting for “catch-up sleep”. Higher scores indicated more of an evening preference. The midpoint of sleep has been shown to correlate strongly with sleep logs, actigraphy and dim-light melatonin onset (Jankowski, 2015; Kantermann et al., 2015; Wright et al., 2013) as it pertains to the circadian rhythm preference.

Depression symptoms were measured via youth self-report with the **Children’s Depression Inventory 2 Short-Form** (CDI-2 SF; (Kovacs & Multi-Health Systems Staff, 2011)), a widely-used, valid (Bae, 2012) self-report questionnaire to measure depression symptoms in youth. The CDI-2 SF contains 12 items instructing youth to identify one of three statements that was most similar to themselves over the previous 2 weeks (e.g., 0 = I am sad once in a while to 2 = I am sad all the time), with higher total scores indicating more elevated symptoms of depression. Internal consistency for the CDI-2 SF has been shown to be between 0.66 and 0.82 (Cumba-Avilés, 2020; Kim et al., 2014). For the current sample, Cronbach alpha ranged between 0.73 to 0.84 across all three timepoints.^1^

The **Pubertal Development Scale** (PDS; Petersen et al., 1988) assessed pubertal development via self-report. Individuals were asked about three characteristics (height, body hair and skin changes) related to pubertal development. Girls were additionally asked about breast development and menstruation, while boys were asked about changes in voice and growth of facial hair. Each characteristic was rated on a 4-point scale, with higher scores indicating more mature pubertal development (i.e., 1 = *has not begun,* 2 = *has barely started*, 3 = *is definitely under way*, 4 = *growth or development is complete*). The exception to the 4-point scale is item 5, inquiring about the start of menarche (“*Have you begun to menstruate (started your period)?”*; 1 = *No,* and 4 = *Yes*). Reliability and validity of the PDS is reported to be moderate to high (Cronbach’s alpha range: 0.66—0.85) and has been shown to converge across self- and physician-rated Tanner stages (Chan et al., 2010; Gau & Soong, 2003; Petersen et al., 1988; Schmitz et al., 2004; Shirtcliff et al., 2009). Within the current sample, Cronbach’s alpha ranged between 0.66 to 0.77.

### Data Analytic Plan

All aims and analyses were pre-registered on the Open Science Framework (OSF) (https://osf.io/s2rq8/). Analyses examining study aims were conducted according to the following procedure: (1) First, chronotypal timing was operationalized as the standardized residuals (the regression of chronotype on age), reflecting discrepancies between observed and expected status scores (following the same procedure outlined by Dorn et al., 2003, 2006 to calculate pubertal timing); (2) next, concurrent associations between chronotypal timing and depressive symptoms were examined; (3) then, chronotypal timing was examined as a predictor of prospective changes in symptoms of depression 6- and 12-months later. Finally, analyses were repeated controlling for pubertal timing, race and ethnicity. For all models, the *unstandardized* effect size is reported. This is to facilitate interpretation due to the fact that the standardized residuals were used in creating the timing variables (i.e., pubertal & chronotypal timing). Therefore the interpretation of the effects are such that one standard deviation in the timing variable is related to *beta* units increase on the CDI-2 SF. R (R Core Team, 2021) and R-Studio (RStudio Team, 2020) were used for the statistical analyses along with the following R packages: ‘here’ (Müller & Bryan, 2020), ‘dplyr’ (Wickham et al., 2023a), ‘psych’ (Revelle, 2023), ‘purr’ (Wickham et al., 2023b), ‘tidyr’ (Wickham et al., 2023c), ‘ggplot2’ (Wickham et al., 2009, ‘sjPlot’ (Lüdecke et al., 2023), ‘ggstatsplot’ (Patil & Powell, 2023), and ‘faux’ (DeBruine et al., 2023).

#### Operationalizing Pubertal and Chronotypal Timing

Calculations for the developmental markers pubertal and chronotypal timing were conducted using the approach outlined by Dorn and colleagues (Dorn et al., 2003, 2006). Specifically, Dorn and colleagues (2003, 2006) operationalized a “timing” variable through the use of standardized residual scores. For the case of pubertal and chronotypal timing, the respective measures (i.e., PDS, MSF & MSF_sc_) were separately regressed on age, and the standardized residuals from these analyses were then saved to reflect the timing variable. The regression including pubertal status and age was performed separately by gender to allow for the standardized residual scores to reflect a comparison to similarly aged and gendered peers (Dorn et al., 2003, 2006). The regression for chronotype and age was not separated by gender to allow for the standardized residual scores to only reflect a comparison to similarly aged peers. Therefore, for chronotypal timing, positive residual scores identified youth who reported more of a preference for evening/late in comparison to same-aged peers (i.e., advanced chronotypal timing), whereas negative residual scores reflected youth showing more of a preference for morning/early when compared to similarly-aged peers (i.e., delayed chronotypal timing). For pubertal timing positive residual scores reflect youth who exhibit a more advanced pubertal status when compared to same-aged and gendered peers, whereas negative residual scores identify youth with a delayed pubertal status compared to similarly-aged and gendered peers.

## Results

### Preliminary Results

Tables 1 and 2 report descriptive statistics and correlations for youth age, chronotype, pubertal status, measures of timing and depression at all three timepoints. Chronotype (MSF & MSF_sc_) measurements were consistent with prior research in a similar age group (Inderkum & Tarokh, 2018) and corresponded to a slight to moderate level of an evening preference (Kühnle, 2006; Suh et al., 2017). Across all three timepoints, chronotype became later, consistent with established normative developmental trends (Carskadon, 2011; Carskadon et al., 1993; Crowley et al., 2018; Roenneberg et al., 2004). Gender differences among variables emerged for pubertal status at all three timepoints (T1: *t* = −6.14, *p* < 0.001; T2: *t* = −6.45, *p* < 0.001; T3: *t* = −7.37, *p* < 0.001) and depressive symptoms at T2 (*t* = −2.17, *p* = 0.032) with girls being more developmentally advanced and showing elevated symptoms of depression (T2). Additionally, sleep corrected chronotype and chronotypal timing (using MSF_sc_) showed gender differences at T3 (*t* = 2.12, *p* = 0.036 and *t* = 2.16, *p* = 0.033, respectively) such that boys had a greater preference for eveningness and an earlier chronotypal timing (i.e., a greater evening preference compared to age). There were no other gender differences (*p’s* > 0.12). There were significant relationships that emerged between age and chronotype (MSF) such that as age increased, chronotype became later (T1: *r* = 0.22, T3: 0.23; *p’s* < 0.05). Symptoms of depression at T1 were related to chronotypal timing (with MSF_sc_) at T2 (*r* = 0.185, *p* < 0.05), while pubertal timing was concurrently related to chronotypal timing (with MSF) at T1 (*r* = 0.173, *p* < 0.05).

**Table 1:** Descriptive statistics

	*Mean*	*SD*	*min*	*max*
Age T1	12.19	0.89	10.00	14.00
Age T2	12.69	0.94	11.00	15.00
Age T3	13.14	0.89	11.00	15.00
PDS T1	11.64	3.41	5.00	19.00
PDS T2	12.51	3.54	5.00	19.00
PDS T3	13.55	3.67	5.00	20.00
MSF T1	4.34	1.23	1.50	7.83
MSF T2	4.75	1.36	1.37	8.00
MSF T3	5.04	1.43	1.58	9.00
MSF-sc T1	3.84	1.21	1.42	7.21
MSF-sc T2	4.14	1.39	1.37	7.88
MSF-sc T3	4.54	1.43	1.00	8.00
CDI T1	4.13	3.24	0.00	17.00
CDI T2	4.10	3.92	0.00	18.00
CDI T3	5.11	4.22	0.00	20.00

**Table 2:** Correlation Table

	Age T1	MSF T1	MSF T2	MSF T3	MSF-sc T1	MSF-sc T2	MSF-sc T3	CDI T1	CDI T2	CDI T3	Pub. Timing T1	Pub. Timing T2	Pub. Timing T3	MSF Timing T1	MSF Timing T2	MSF Timing T3	MSF-sc Timing T1	MSF-sc Timing T2
MSF T1	0.222**																	
MSF T2	0.087	0.623***																
MSF T3	0.228*	0.585***	0.548***															
MSF-sc T1	0.149	0.911***	0.623***	0.505***														
MSF-sc T2	0.048	0.543***	0.918***	0.475***	0.625***													
MSF-sc T3	0.138	0.518***	0.519***	0.926***	0.489***	0.503***												
CDI T1	0.071	0.091	0.178	0.063	0.035	0.195*	0.080											
CDI T2	0.038	−0.082	−0.001	−0.006	−0.092	0.027	−0.030	0.622***										
CDI T3	0.085	0.049	0.056	0.009	0.041	0.055	−0.047	0.609***	0.650***									
Pub. Timing T1	−0.001	0.163	−0.104	−0.084	0.144	−0.044	−0.094	0.106	0.132	0.138								
Pub. Timing T2	0.115	0.166	0.009	0.038	0.085	0.015	−0.008	0.053	0.047	0.175*	0.657***							
Pub. Timing T3	0.008	0.131	−0.009	0.021	0.009	−0.043	−0.057	0.118	0.051	0.142	0.538***	0.694***						
MSF Timing T1	−0.000	0.975***	0.621***	0.552***	0.897***	0.549***	0.504***	0.073	−0.097	0.033	0.173*	0.147	0.137					
MSF Timing T2	−0.018	0.606***	0.993***	0.521***	0.618***	0.915***	0.504***	0.165	−0.003	0.056	−0.102	0.014	−0.012	0.628***				
MSF Timing T3	−0.034	0.526***	0.537***	0.964***	0.461***	0.466***	0.908***	0.029	−0.031	−0.023	−0.101	0.005	0.016	0.556***	0.541***			
MSF-sc Timing T1	−0.000	0.890***	0.614***	0.479***	0.989***	0.623***	0.477***	0.022	−0.101	0.030	0.152	0.070	0.009	0.910***	0.627***	0.476***		
MSF-sc Timing T2	−0.032	0.529***	0.908***	0.455***	0.620***	0.996***	0.491***	0.185*	0.028	0.056	−0.042	0.020	−0.044	0.554***	0.918***	0.470***	0.632***	
MSF-sc Timing T3	−0.037	0.472***	0.508***	0.896***	0.455***	0.495***	0.984***	0.056	−0.046	−0.069	−0.108	−0.028	−0.061	0.499***	0.513***	0.926***	0.470***	0.499***

*Computed correlation used pearson-method with pairwise-deletion*

Using Little’s MCAR test (Little, 1988), the data were found to not be missing completely at random (MCAR; p < 0.05). To further investigate missingness, variables of interest with the current study were examined to see if there were statistically significant differences between participants who completed the study versus those who did not (Enders, 2022). Participants who did not complete the T2 assessment showed slightly later chronotype – not corrected for sleep debt (4.91) than those who did (4.2; t = 2.94, *p* = *0.005*). All other differences were not significantly different among missingness patterns (*p’s* > 0.07). Therefore, missing patterns were assumed to be missing at random and we proceeded using the default method within the linear regression function in R (case wise deletion).

#### Chronotypal Timing and Depression

Chronotypal timing (not corrected for catch-up sleep; using MSF) significantly predicted prospective changes in depressive symptoms in the 6-months from T1 to T2 (*b* = −0.66, CI [−1.20 – −0.11], *p* = 0.019; Table 3), but did not significantly predict changes in depressive symptoms from T2 to T3 (*b* = 0.16, CI [−0.43 – 0.75], *p* = 0.72). Chronotypal timing was not significantly related to concurrent levels of depression (*b* = 0.24, CI [−0.31 – 0.79], *p* = 0.40), nor prospectively predictive of changes symptoms of depression 12-months later (*b* = −0.13, CI [−0.74 – 0.49], *p* = 0.69). Chronotypal timing continued to predict prospective changes in symptoms of depression (T1 to T2) while controlling for gender, race and ethnicity (*b* = −0.63, CI [−1.18 – −0.09], *p* = 0.022). All other findings showed a similar pattern (Supplementary Table 1).

**Table 3:** Chronotypal timing (Not Sleep Corrected) & depression

	Concurrent (T1)			6-Months (T1- > T2)			12-Months (T1- > T3)			6-Months (T2- > T3)		
	*Estimates*	*CI*	*p-value*	*Estimates*	*CI*	*p-value*	*Estimates*	*CI*	*p-value*	*Estimates*	*CI*	*p-value*
Intercept	4.22	3.67 – 4.78	** < 0.001**	0.99	0.11 – 1.86	**0.028**	1.76	0.79 – 2.74	**0.001**	2.49	1.60 – 3.37	** < 0.001**
Chron. Timing-T1	0.24	−0.31 – 0.79	0.396	−0.66	−1.20 – −0.11	**0.019**	−0.13	−0.74 – 0.49	0.686			
CDI-T1				0.78	0.62 – 0.95	** < 0.001**	0.81	0.63 – 1.00	** < 0.001**			
Chron. Timing-T2										0.16	−0.43 – 0.75	0.584
CDI-T2										0.62	0.47 – 0.78	** < 0.001**
Observations	138			130			125			112		
R^2^ / R^2^ adjusted	0.005 / −0.002			0.417 / 0.407			0.396 / 0.386			0.375 / 0.363		

When accounting for sleep debt accumulated through the week (i.e., “catch-up sleep”), chronotypal timing (using MSF_sc_) was not significantly associated with concurrent depression symptoms (*b* = 0.07, CI [−0.49 – 0.64], *p* = 0.80) (Table 4). This conceptualization of chronotypal timing did not significantly predict prospective changes in symptoms of depression 6-months (T1 to T2: *b* = −0.51, CI [−1.08 – 0.06], *p* = 0.08; T2 to T3: *b* = 0.11, CI [−0.49 – 0.71], *p* = 0.72) or 12-months later (*b* = 0.03, CI [−0.59 – 0.66], *p* = 0.92) after controlling for the previous timepoints levels of depressive symptoms. Results suggest that chronotypal timing, when corrected for catch-up sleep, is not related to concurrent or prospective symptoms of depression. A similar patterning was found when controlling for gender, race and ethnicity (*p’s* > 0.099; Supplementary Table 2).^2^

**Table 4:** Chronotypal timing (Sleep Corrected) & depression

	Concurrent (T1)			6-Months (T1- > T2)			12-Months (T1- > T3)			6-Months (T2- > T3)		
	*Estimates*	*CI*	*p-value*	*Estimates*	*CI*	*p-value*	*Estimates*	*CI*	*p-value*	*Estimates*	*CI*	*p-value*
Intercept	4.25	3.69 – 4.82	** < 0.001**	1.13	0.22 – 2.04	**0.015**	1.82	0.83 – 2.82	** < 0.001**	2.48	1.56 – 3.39	** < 0.001**
Chron. Timing-T1	0.07	−0.49 – 0.64	0.798	−0.51	−1.08 – 0.06	0.077	0.03	−0.59 – 0.66	0.916			
CDI-T1				0.76	0.59 – 0.92	** < 0.001**	0.81	0.62 – 0.99	** < 0.001**			
Chron. Timing-T2										0.11	−0.49 – 0.71	0.722
CDI-T2										0.62	0.47 – 0.78	** < 0.001**
Observations	134			126			122			108		
R^2^ / R^2^ adjusted	0.000 / −0.007			0.396 / 0.387			0.390 / 0.380			0.378 / 0.366		

### Chronotypal & Pubertal Timing

To examine the relative specificity of effects to chronotypal timing compared with other salient co-occurring developmental processes (i.e., pubertal timing), we conducted a series of follow-up analyses in which pubertal timing was included as a covariate in tests of primary hypotheses. Controlling for pubertal timing, chronotypal timing (not corrected for catch-up sleep; using MSF) remained a significant predictor of prospective changes in depressive symptoms in the 6-months from T1 to T2 (*b* = −0.72, CI [−1.27 – −0.16], *p* = 0.012) (Table 5). There were no other statistically significant timing variables (chronotypal or pubertal) that predicted symptoms of depression. When controlling for gender, race and ethnicity, a similar pattern emerged such that chronotypal timing continued to predict changes in symptoms of depression between T1 and T2 (*b* = −0.70, CI [−1.25 – −0.15], *p* = 0.013) beyond the impact of pubertal timing (Supplementary Table 3).

**Table 5:** Chronotypal (NOT sleep corrected) and pubertal timing & depression

	Concurrent (T1)			6-Months (T1- > T2)			12-Months (T1- > T3)			6-Months (T2- > T3)		
	*Estimates*	*CI*	*p-value*	*Estimates*	*CI*	*p-value*	*Estimates*	*CI*	*p-value*	*Estimates*	*CI*	*p-value*
Intercept	4.24	3.68 – 4.80	** < 0.001**	0.99	0.11 – 1.87	**0.029**	1.86	0.87 – 2.84	** < 0.001**	2.61	1.73 – 3.48	** < 0.001**
Chron. Timing-T1	0.18	−0.39 – 0.75	0.540	−0.72	−1.27 – −0.16	**0.012**	−0.23	−0.85 – 0.40	0.473			
Pub. Timing-T1	0.28	−0.29 – 0.84	0.331	0.46	−0.07 – 0.99	0.091	0.50	−0.09 – 1.09	0.096			
CDI-T1				0.78	0.61 – 0.94	** < 0.001**	0.80	0.62 – 0.98	** < 0.001**			
Chron. Timing-T2										0.15	−0.44 – 0.73	0.617
Pub. Timing-T2										0.70	0.12 – 1.28	**0.018**
CDI-T2										0.61	0.45 – 0.76	** < 0.001**
Observations	136			128			123			111		
R^2^ / R^2^ adjusted	0.012 / −0.003			0.436 / 0.422			0.410 / 0.395			0.407 / 0.390		

When accounting for catch-up sleep, chronotypal timing (using MSF_sc_) was found to significantly predict changes in symptoms of depression between T1 and T2 after taking pubertal timing into account (*b* = −0.57, CI [−1.14 – −0.002], *p* = 0.049) (Table 6). The confidence interval for this effect was very close to zero, suggesting that this may not be a robust and replicable effect. Similar to the previous models, no other timing variable was statistically significant. However, when controlling for gender, race and ethnicity, chronotypal timing (corrected for catch-up sleep) was no longer a significant predictor of changes in symptoms of depression (*b* = −0.54, CI [−1.11 – −0.03], *p* = 0.063)(Supplementary Table 4). Findings from these models suggest that chronotypal timing (not correcting for catch up sleep) may play a particularly important role as conferring risk for changes in depression, above and beyond other co-occurring developmental processes.

**Table 6:** Chronotypal (sleep corrected) and pubertal timing & depression

	Concurrent (T1)			6-Months (T1- > T2)			12-Months (T1- > T3)			6-Months (T2- > T3)		
	*Estimates*	*CI*	*p-value*	*Estimates*	*CI*	*p-value*	*Estimates*	*CI*	*p-value*	*Estimates*	*CI*	*p-value*
Intercept	4.28	3.71 – 4.85	** < 0.001**	1.15	0.23 – 2.06	**0.014**	1.93	0.92 – 2.93	** < 0.001**	2.55	1.65 – 3.45	** < 0.001**
Chron. Timing-T1	0.01	−0.57 – 0.59	0.969	−0.57	−1.14 – −0.00	**0.049**	−0.05	−0.69 – 0.58	0.874			
Pub. Timing-T1	0.36	−0.22 – 0.94	0.219	0.49	−0.06 – 1.04	0.083	0.51	−0.09 – 1.12	0.093			
CDI-T1				0.75	0.58 – 0.92	** < 0.001**	0.79	0.60 – 0.97	** < 0.001**			
Chron. Timing-T2										0.09	−0.50 – 0.68	0.772
Pub. Timing-T2										0.70	0.11 – 1.29	**0.022**
CDI-T2										0.61	0.46 – 0.76	** < 0.001**
Observations	132			124			120			107		
R^2^ / R^2^ adjusted	0.012 / −0.003			0.418 / 0.404			0.404 / 0.388			0.409 / 0.392		

### Exploratory Analyses

#### Predicting Chronotype

Previous work has identified a reciprocal relationship between symptoms of depression and chronotype (e.g., Haraden et al., 2017). Exploratory analyses therefore evaluated potential reciprocal relations between depression and chronotypal timing. Symptoms of depression were used to predict later individual differences of chronotypal timing (as measured with MSF or ${\text{MSF}}_{\text{sc}}$) controlling for pubertal timing, gender, race and ethnicity identical to the previous analyses. Controlling for pubertal timing, symptoms of depression did not significantly predict concurrent or prospective changes in chronotypal timing (MSF—*not* sleep corrected)(Supplementary Table 5).symptoms of depression at T1 significantly predicted changes in chronotypal timing (${\text{MSF}}_{\text{sc}}$—corrected for sleep debt) from T1 to T2 (*b* = 0.06, CI [0.01 – 0.10], *p* = 0.018)(Supplementary Table 6). These results remained significant after controlling for gender, race and ethnicity (Supplementary Table 7), although the effect continues to be rather small (*b* = *0.05,* CI [0.01 – 0.10], *p* = 0.03).

#### Gender Interaction Effects

Exploratory gender moderation analyses were conducted to examine potential gender differences in associations between chronotypal timing and depressive symptoms from previous models. The main findings presented above were further examined by including an interaction term between gender and the respective chronotypal timing variable. Gender did not significantly moderate associations between chronotypal timing (MSF or ${\text{MSF}}_{\text{sc}}$) and concurrent or prospective symptoms of depression (Supplementary Table 8).

## Discussion

The period of development from childhood into adolescence consists of physical/biological, social and psychological transitions and is a period of increased rates of psychopathology, including depression among youth (e.g., Avenevoli et al., 2001; Hankin et al., 2015; Kessler et al., 2005; Merikangas et al., 2010). It has become increasingly important to expand investigations into mechanisms and risk factors beyond mean-level differences and to examine timing of these various processes. In an effort to account the developmental intricacies involved in even the most normative and universal biological processes, the current manuscript was the first to investigate effects of the *timing* of the development of chronotypal on concurrent and prospective depression. Chronotypal timing represents a novel means by which to operationalize the relative timing of an individual’s transition in circadian rhythm across late childhood and adolescence (Roenneberg et al., 2004). The present findings indicate that youth vary in their circadian timing, with implications for future depression symptoms. Thus, results suggest that a focus on the *timing* of chronotypal development, in addition to mean-level differences in chronotype, is essential to advance a more nuanced understanding of the role of circadian processes in adolescent risk for psychopathology.

The present study advances knowledge by proposing and examining a conceptual and statistical model capturing *chronotypal timing* during adolescence. Chronotypal timing, conceptualized as the extent to which youth chronotype development unfolds in tandem with same-aged peers and modeled using a standardized residual approach, predicted symptoms of depression 6-months later (T1 → T2) after controlling for baseline levels of depression and gender. Importantly, this relationship appeared to be specific to chronotypal timing when measured *without* correcting for catch up sleep. Results suggest that youth who experience *late chronotypal timing* (not corrected for catch up sleep) relative to their peers (i.e., more morningness oriented relative to same-aged peers) are at increased risk for later elevations in symptoms of depression. This may highlight a disparity between youth’s developing social identities and the opportunities that they have to connect with others. During this time, youth often seek distance from their parents and to cultivate closer interpersonal relationships with peers (Klimstra et al., 2010; Meeus et al., 2005). Thus, maintaining an earlier chronotype compared to similarly aged peers may result in missing important peer socialization opportunities, ultimately conferring risk for depression. The results also hold a wide confidence interval around the estimate, suggesting additional investigation into the conceptualization of chronotypal timing may be needed (i.e., in relation to other developmental processes). Chronotypal timing was not significantly related to symptoms of depression at baseline, 12-months later or the 6-month interval between T2 and T3. This may suggest a limited window of impact for chronotypal timing upon youth’s symptoms of depression. Chronotypal timing may likely have different levels of impact upon an individual at varying timescales. It will be important for future research to be able to identify the amount of time in which chronotypal timing will have the largest influence upon youth’s well-being.

The main results of this study showing that chronotypal timing (*not* sleep corrected) predicted later changes in depressive symptoms across six months were retained when accounting for pubertal timing, an additional, highly important co-occurring developmental process. Additionally, when including pubertal timing in the model, chronotypal timing (corrected for sleep debt) became a significant predictor of changes in symptoms of depression 6-months later. These findings may suggest that pubertal timing is a suppressor variable within this model. Including both constructs into the analysis is important and it allowed for a more holistic examination of this developmental time-period and promoted an integration between the “social zeitgeber” theory (Ehlers, 1988) and the model proposed by Chauhan and colleagues (2023). Findings from the current study highlight that chronotypal timing may play a unique role in changes in symptoms of depression during early adolescence beyond effects of pubertal timing. It will also be important to understand the possibility of reciprocal relationships among these factors and how the developmental patternings unfold over time as well as the integration among other social processes (e.g., peer and interpersonal relationships). Further investigation of chronotypal timing may continue to enrich our understanding of the integration between biological and social processes during late childhood and into adolescence.

Within the current study, the construct of chronotype/chronotypal timing was calculated using two different methods following procedures by Roenneberg and colleagues (2003, 2012, 2019): midpoint of sleep on free days (MSF) and midpoint of sleep on free days – corrected for sleep debt (MSF_sc_). Originally, MSF had been indicated as the metric by which chronotype was categorized due to midsleep being reported as an anchor point for melatonin onset (Terman et al., 2001), and “free days” being chosen as they are not constrained by social/occupational/academic endeavors, allowing the internal clock to be unimpeded (Roenneberg et al., 2003; Zavada et al., 2005). However, sleep duration is drastically different between free days and work/school days, with particularly notable discrepancies among individuals with later chronotypes (Roenneberg et al., 2007). Measures of chronotype (MSF) were in need of a correction for accumulated sleep debt between short sleep durations during the week and compensatory sleep during free days/weekends, resulting in midpoint of sleep on free days – corrected for sleep debt (MSF_sc_; Roenneberg et al., 2007). MSF_sc_ was then suggested as the metric to be used when evaluating chronotype as it was “uncontaminated” by differences in sleep duration. The results of the current study highlight chronotype/chronotypal timing (MSF), not corrected for sleep debt, as being significantly related to changes in symptoms of depression. By taking the sleep debt into account (MSFsc), chronotype/chronotypal timing is no longer related to depressive symptoms. The current study was able to highlight the differential impact between MSF and MSF_sc_ upon symptoms of depression, suggesting potential mechanisms toward the relationship between chronotype/chronotypal timing and psychopathology.

The discrepancy of sleep duration between school days and free days is particularly salient for youth, with at least half of youth experiencing a difference of 2 h or greater between weekdays and weekend days (Roenneberg et al., 2012; Wittmann et al., 2006). By accounting for (removing) sleep debt in the measure of chronotype/chronotypal timing, we may be removing a portion of the mechanism of dysfunction that is reflected in this construct that elevates risk for symptoms of depression. Including sleep debt within the measure may suggest that the mechanism of focus should be the overall discrepancy among sleep patterns. These findings may suggest updating our conceptualization of risk as it relates to chronotype in an attempt to incorporate additional metrics of inconsistency/discrepancy in sleep patterns (e.g., social jetlag). It may be the case that holding a later chronotype provide greater opportunities for inconsistencies to emerge (high variability in bed/wake times for youth), resulting in various patterns of synchrony and asynchrony. These findings support taking a more holistic approach to examining the role of chronotype as a risk factor suggested by Chauhan, et al. (2023). Future studies should consider examining MSF (not corrected for sleep) and other variables of sleep development to focus on synchrony across these processes. Although previous literature has emphasized sleep debt and catch-up sleep (sleeping in later on free days to account for sleep that had been “missed” during weekdays) have negative impact on well-being (for review see Roenneberg et al., 2019), a large cohort study concluded that individuals with a short sleep duration during the workdays had a higher mortality rate than those who were able to have some catch-up sleep on the weekend/free days (Åkerstedt et al., 2019). Therefore, it will be important for future work to continue to integrate multiple dimensions when investigating chronotype as it relates to the unique developmental time period of adolescence.

### Strengths and Limitations

The current study has a number of strengths and advances knowledge of chronotypal development as it relates to symptoms of depression both concurrently as well as 6 and 12-months later. The age of this sample (i.e., early adolescence) is particularly vulnerable period of development in which several important developmental milestones occur, such as the time in which there tends to be a marked increase in rates of depression (Costello et al., 2003; Hankin et al., 1998), as well as the period in which the transition of chronotype has been previously observed (Roenneberg et al., 2004). Additionally, the study used a prospective design to be able to capture both concurrent and prospective changes in the phenomena of interest.

It is also important to consider limitations within the current study as they offer areas to expand upon in future research. First, the overall sample characteristics were largely homogeneous as it applies to the overall racial and ethnic makeup. Individuals were predominantly white (71.2%) and the majority of the caregivers of the youth had completed at least a 4-year bachelor’s degree or higher (83.9%). Thus, the generalizability of findings to other populations is unclear. The individuals included in the present work may experience a higher level of financial stability which may result in a greater ability for control over their schedules and ultimately reduce any level of unpredictability. In addition, collecting bed and wake times by an open text self-report field introduced variability in terms of the amount of missing data that was present. A portion of individuals responded with a text response when asked about bedtime (e.g., “When I feel tired”), resulting in chronotype being considered missing at that timepoint. Such variability in the missingness may impact the interpretations and future work should implement structured methods to collect that information. It is also important to note that for some youth in the study, the 6- and 12- month follow-up assessments occurred in the context of COVID-19 related restrictions, but previous analyses in this sample indicated no systematic effects of pandemic status on depressive symptom outcomes (Griffith & Hankin, 2024). Additionally, there is a large body of research highlighting the differential impact of pubertal timing across diverse backgrounds. Previous work has shown that early pubertal timing for Caucasian girls and late pubertal timing for African American girls placed them at increased risk for depression (Hamlat et al., 2015). It is unclear to what extent there are differences among race and ethnicities as it pertains to chronotype, but previous research has suggested that Asian American youth may show a greater eveningness compared to their same aged peers (Adam et al., 2007; Fuligni & Hardway, 2006; Karan et al., 2021). Differences in chronotype have also been suggested to be moderated by the parenting styles and the customs within the home (Adam et al., 2007; Fuligni & Hardway, 2006). However, there is a dearth of research examining racial and ethnic differences for chronotype preference, and no study to our knowledge has applied this lens to aspects of chronotypal development and timing. Additionally, it is likely the case that interrelationships and bidirectional effects exist between chronotypal timing and depression.

Future work will need to employ rigorous longitudinal methodologies to properly examine how these relationships unfold over time. Next steps in this field will also incorporate multiple measurement modalities, including youth’s own perceptions of the impact of desynchrony on their well-being. Examining such individual differences will be imperative to providing an accurate understanding of chronotype and developmental synchrony for youth.

## Conclusions

The current study examined the timing of chronotypal development as it relates to concurrent and prospective levels of depression symptoms. These findings suggest that youth who show an earlier chronotype when compared to similarly aged peers (i.e., late chronotypal timing) are at risk for increases in symptoms of depression 6-months later. However, these findings appeared to be specific to the 6-month change from T1 to T2, which may suggest the need to examine these processes earlier in development. That is, the strength of the association between chronotypal timing and depressive symptoms seems to be attenuated among older youth, suggesting that chronotypal timing might be especially relevant prior to mid-adolescence. Further work should aim to replicate these results, as well as examine the longitudinal change in these constructs and their interrelations throughout this unique developmental time period.

## Supplementary Information

Below is the link to the electronic supplementary material.

Supplementary file1 (DOCX 742 KB)

## Data Availability

The participants of this study did not give written consent for their data to be shared publicly, so due to the sensitive nature of the research supporting data is not available.

## Footnotes

1 The CDI-2 SF contains one item that pertains to fatigue. To investigate potential overlap and biased estimates, this item was removed to conduct sensitivity analyses. All patterns of results were identical. Therefore, the results presented here use the complete item list to compute the score for the CDI-2-SF.

2 Additional analyses examining chronotype (and not chronotypal timing) are presented in supplementary tables 9 & 10.
