# Supplementary figures and images for: Temporal Tendencies: Exploring the Impact of Chronotype Timing on Youth Depression Risk
Source: Res Child Adolesc Psychopathol. 2025 Jan 24;53(3):363–79. doi: 10.1007/s10802-024-01287-6 (PMC11913923; doi:10.1007/s10802-024-01287-6)

**Supplementary Materials**

**Regression Tables**

*Chronotypal Timing and Depression*


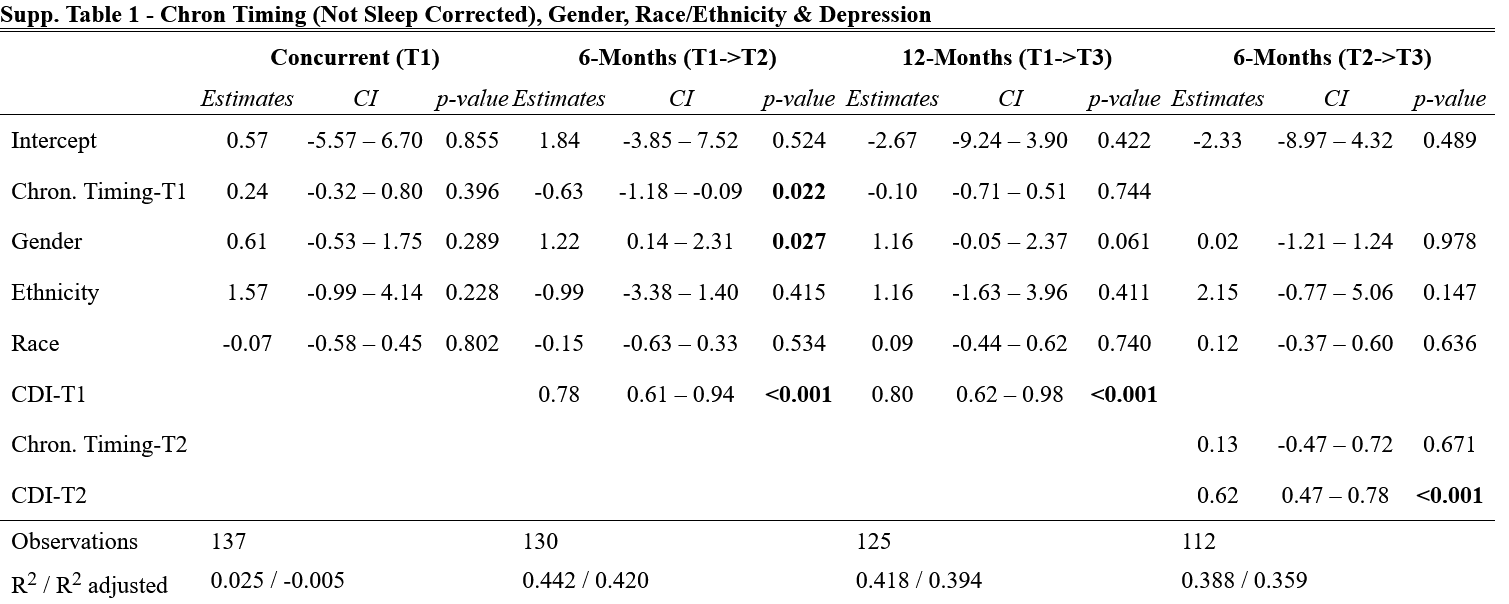


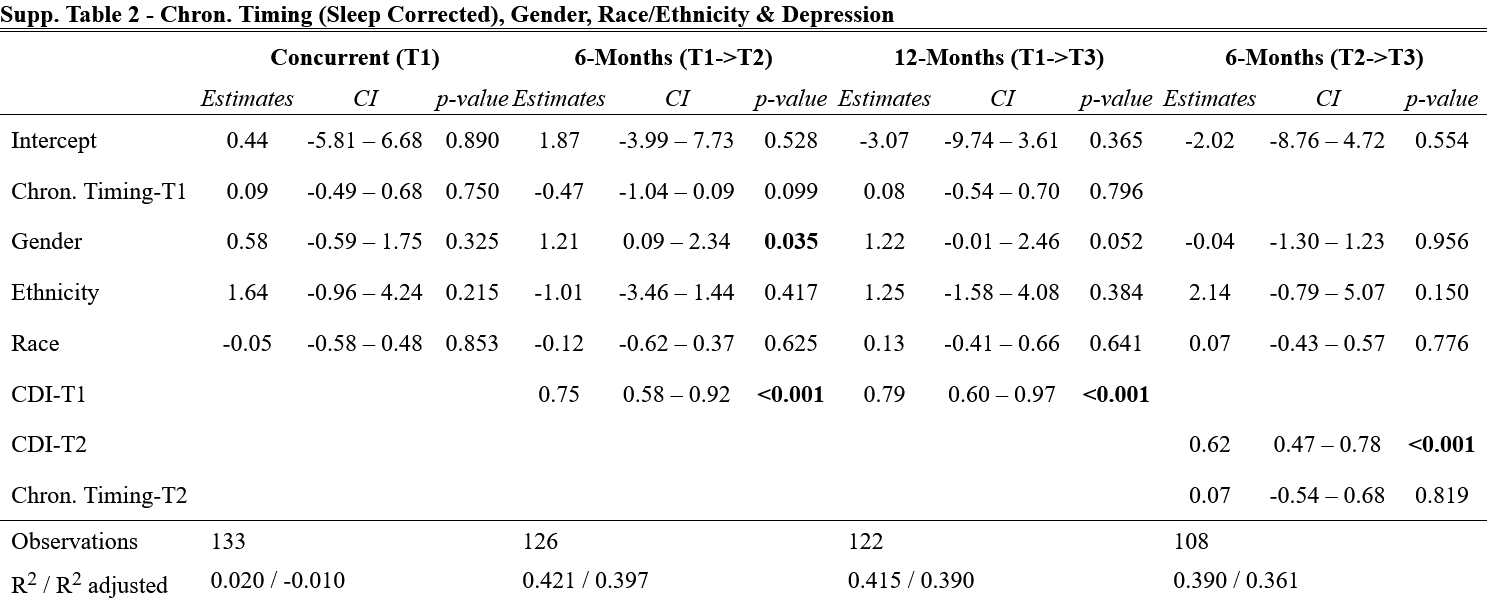


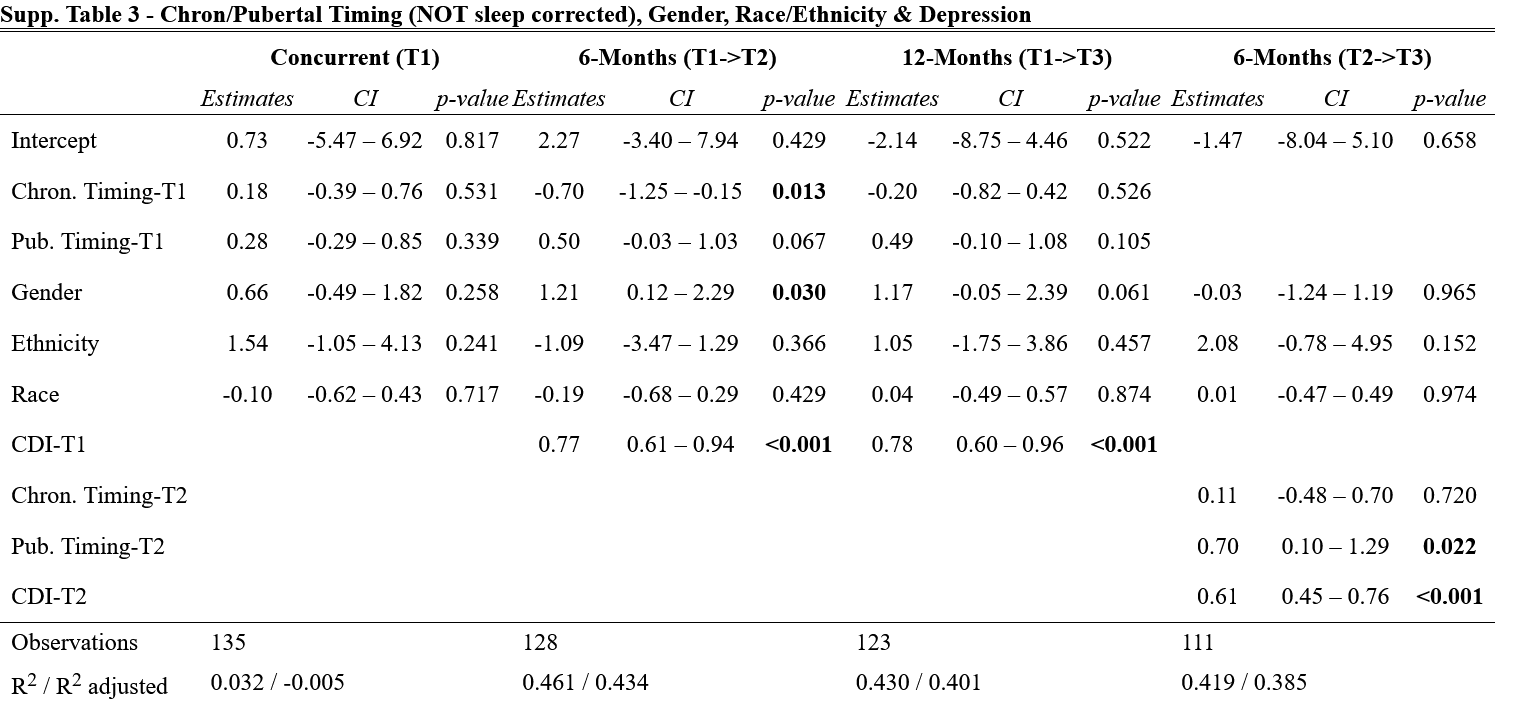


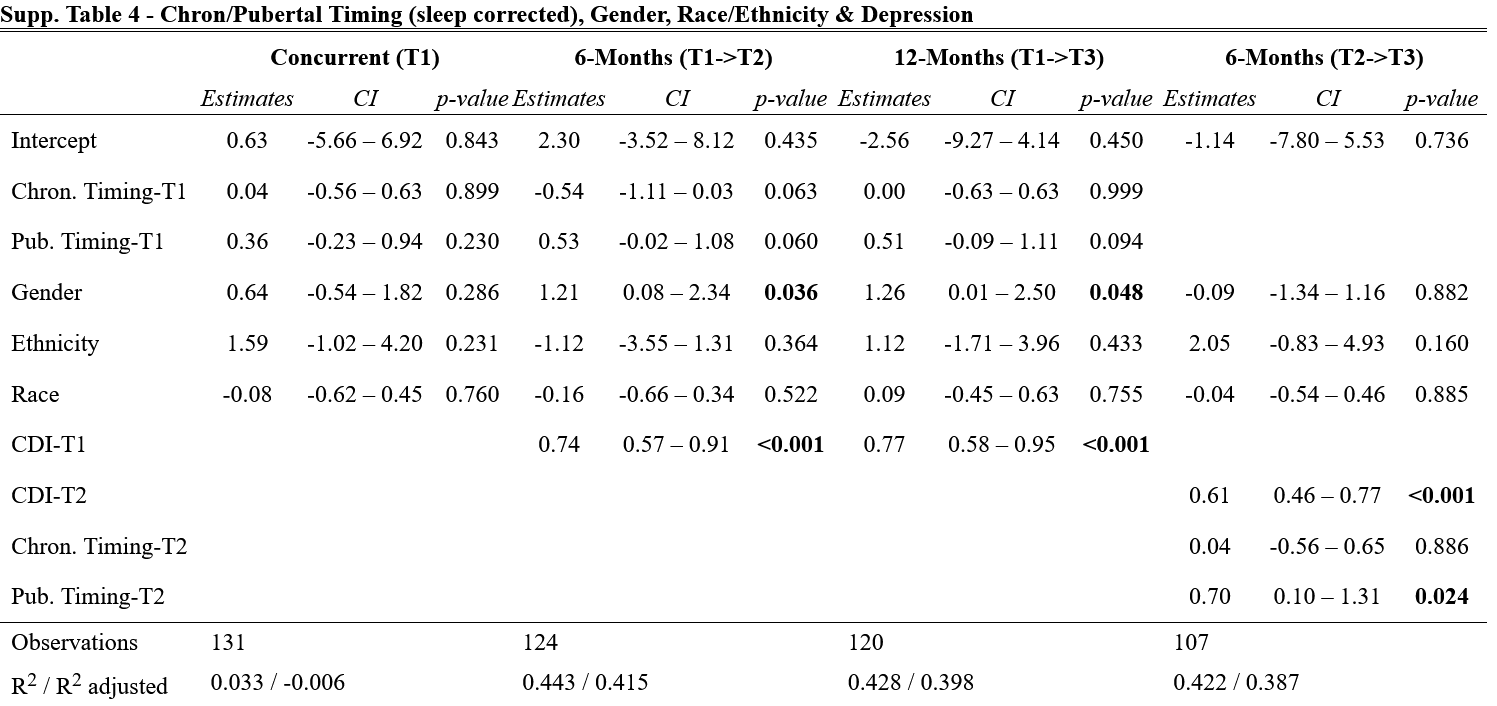


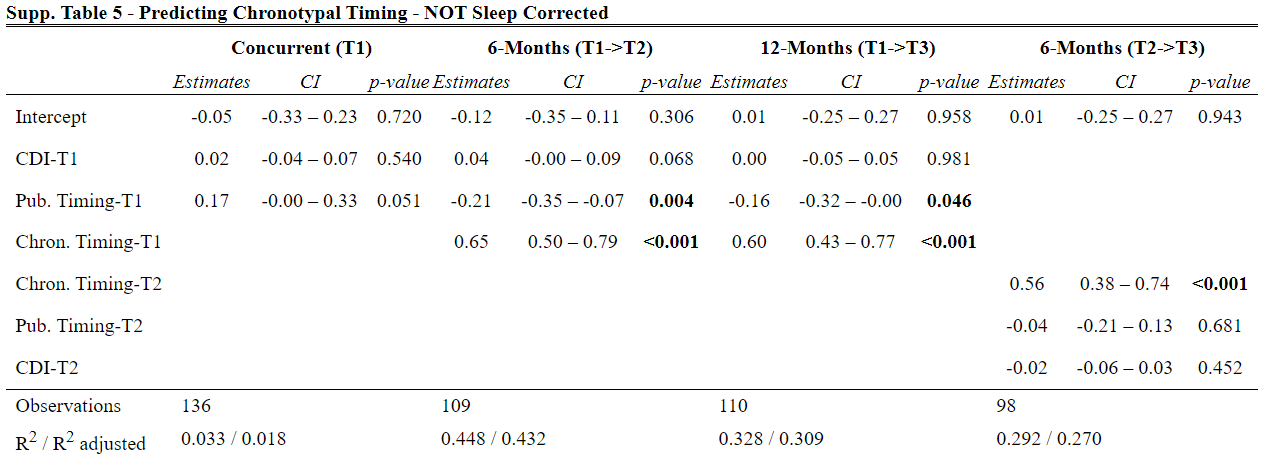


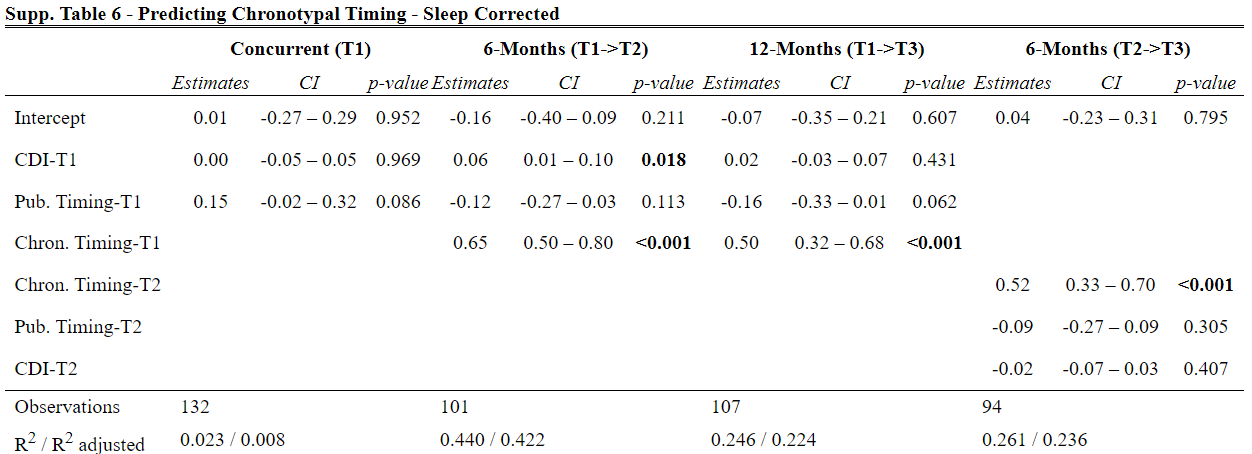


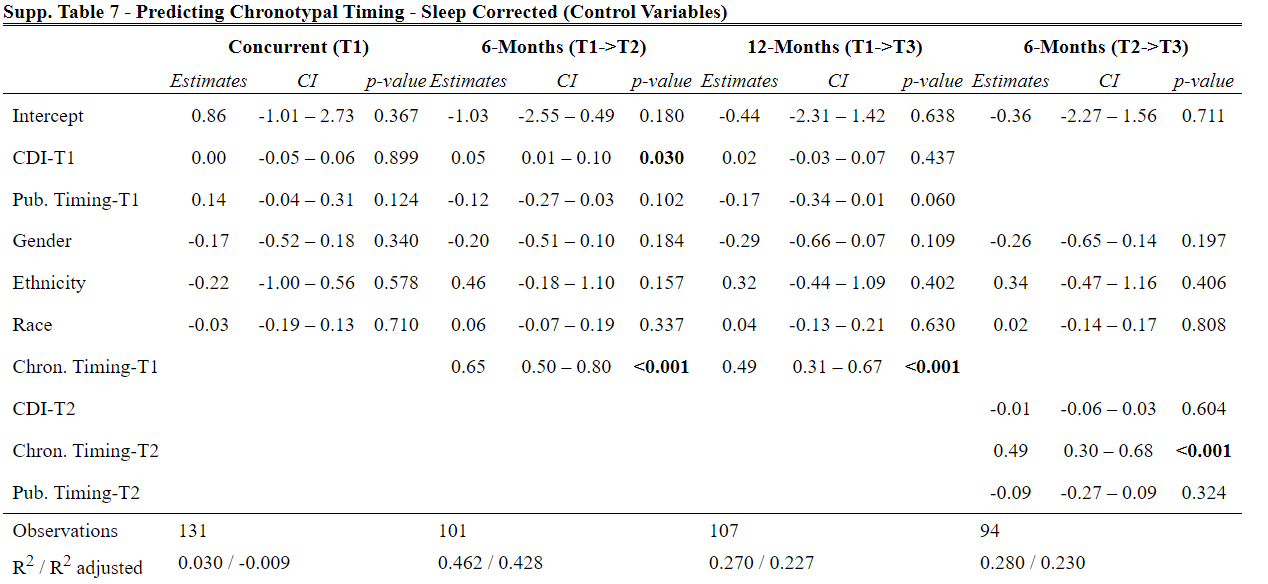


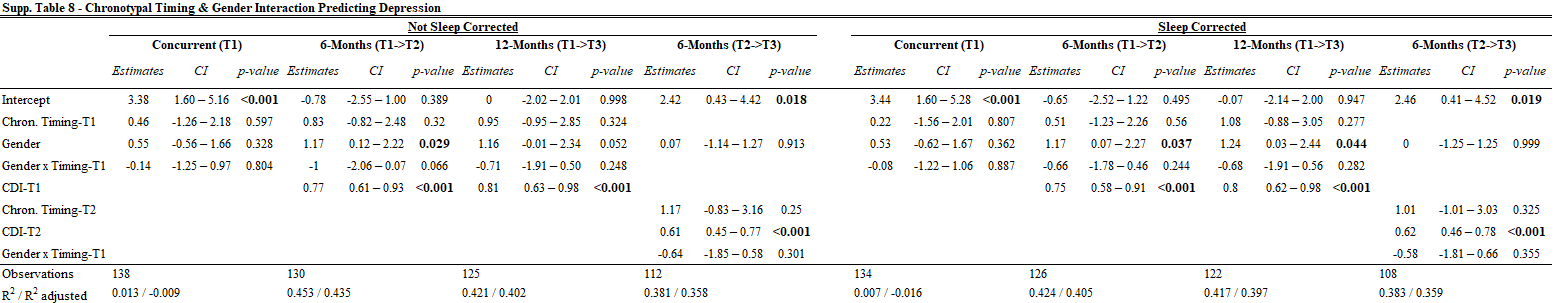


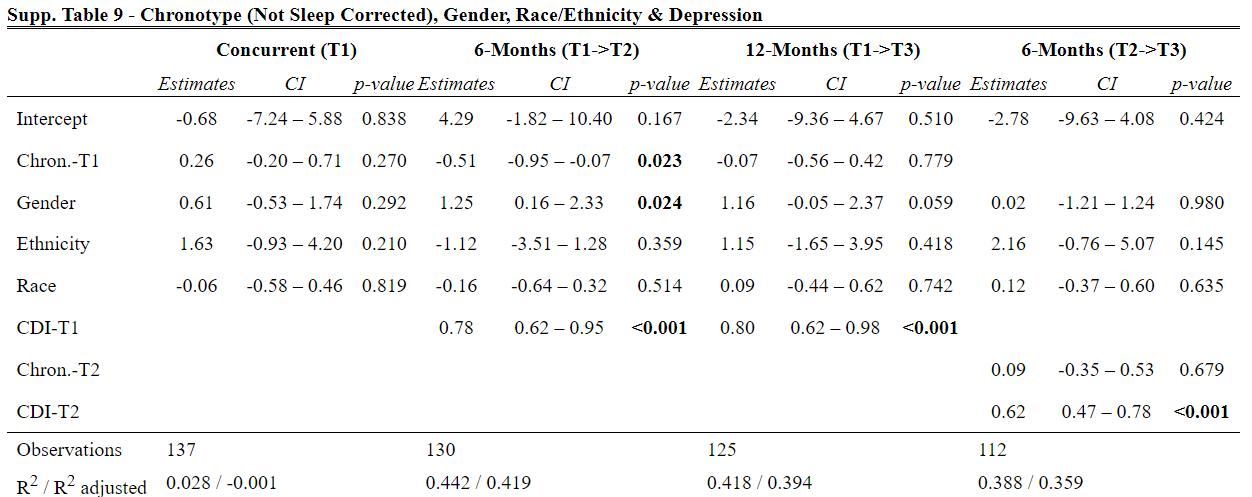


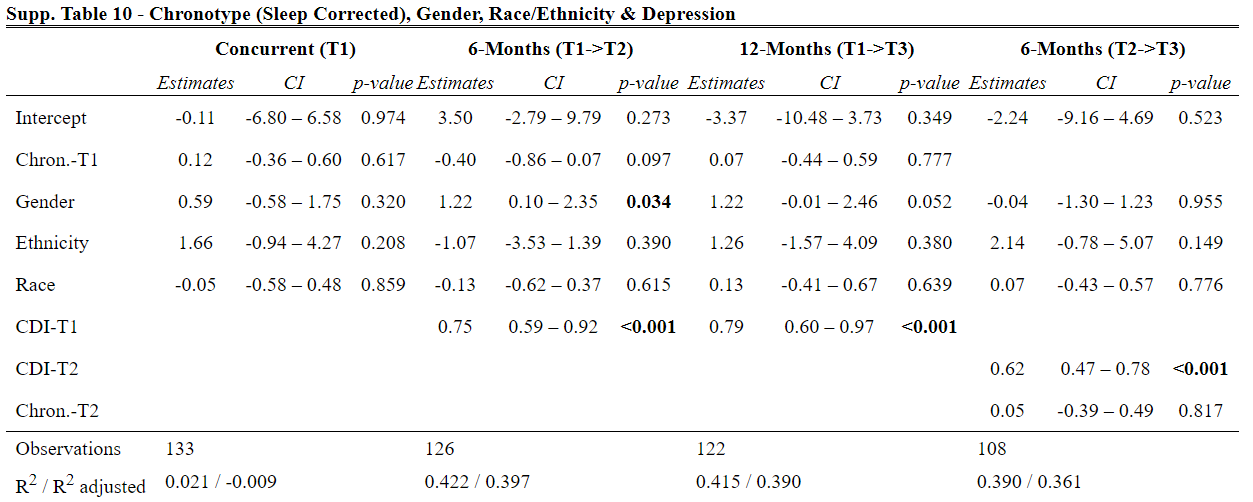

Supplement: Supplementary file 1 — Supplementary file1 (DOCX 742 KB) [file 10802_2024_1287_MOESM1_ESM.docx]
